# Anomalous Origin of the Descendens Noni

**Published:** 1880-03

**Authors:** G. Frank Lydston

**Affiliations:** Charity Hospital, New York


### Clinical Reports.

### NOTES FROM PRIVATE PRACTICE.

### ARTICLE VI.

*Anomalous Origin of the Descendens Noni.*

In a recent dissection of the triangles of the neck, I met with an anomalous origin of the descendens noni, which may prove interesting to anatomists. In the ordinary text books the statement is made that the descendens noni is given off from the ninth or hypoglossal nerve, and joins just below the middle of the neck, to form a loop with the branches from the second and third cervical nerves, known as the communicans noni, lying upon the carotid sheath or occasionally within it. This being the rule, the case in question presents a wide variation from it. On opening the carotid sheath, I found a nerve corresponding to the descendens noni as above described, but on tracing it carefully upward found that it became intimately blended with the pneumogastric nerve, and could not be traced farther without separation of the fibers of the latter. It lay upon the internal jugular vein, crossing it obliquely, and piercing the carotid sheath in its lower third, to be distributed to the muscles of the anterior region of the neck, *i. e.*, the depressors of the os hyoides. The communicating branches from the second and third cervical, ordinarily known as the communicans noni, were absent.

As far as I have been able to investigate, there are no cases recorded, which are precisely similar to the above. Heule describes this nerve, as "nervus cervicalis descendens," which is composed of the "ramus descendens" from the hypoglossal, and branches from the second or a loop between the second and third cervical nerves, which correspond to the communicans noni of Gray and others.

Höll has demonstrated that the branch which apparently proceeds from the hypoglossal, is composed of fibers ultimately derived from the first or second cervical nerves. In some of the old text-books, there is a statement that the ramus descendens, or portion from the hypoglossal really takes its origin in the "ganglioform plexus of the vagus," which corresponds to the "ganglion inferius," instead of the ninth. This variety, Heule states, is more correctly described, according to C. Krause, as the passage of the "ramus descendens" into the neurilemma of the vagus, whence comes the appearance as if it took its origin from the same, adding, however, the statement that cases may happen in which the ramus descendens from the ninth is entirely absent, and in which the branches to the depressors of the os hyoides are derived directly from the par vagum; quoting as authorities Pye Smith, Howse and Davies Colley. (Guy's Hospital Rep. III, Series XVI, 161). Luschka states that the descendens noni, usually contains no filaments from the hypoglossal, but is a branch from the first and second cervical nerves temporarily associated with the ninth. This would agree with the circumstance that in some animals those muscles which are supplied by the branches of the descendens noni in the human subject, receive their nervous supply from the cervical plexus.

In a case of Turner's (Journal of Anatomy VI, 102), the ramus descendens was derived from the vagus and afterward sent a portion of its fibers back again to the trunk of that nerve.

The case that I have above described adds another to the instances of anomalous origin which have been recorded, and appears to differ from them all in the fact of the absence of the communicans noni. The ultimate origin may have been the same as in those examples, in view of the communications which exist between the hypoglossal, vagus and superior cervical nerves, at the "plexus ganglioformis," which is merely a spreading out of the fibers of the vagus by the interposition of adipose connective tissue, this arrangement giving rise to a ganglioform enlargement commonly termed the "ganglion of the trunk," or "ganglion inferius" of the vagus.

It was impossible to dissect out of this structure with its filaments of communication with any degree of accuracy, on account of the large amount of adipose tissue surrounding the part, and the exceedingly poor state of preservation of the subject.

G. FRANK LYDSTON, M.D.

CHARITY HOSPITAL, NEW YORK.

